# Demographic History and Reproductive Output Correlates with Intraspecific Genetic Variation in Seven Species of Indo-Pacific Mangrove Crabs

**DOI:** 10.1371/journal.pone.0158582

**Published:** 2016-07-05

**Authors:** Sara Fratini, Lapo Ragionieri, Stefano Cannicci

**Affiliations:** 1 Department of Biology, University of Florence, Firenze, Italy; 2 Institute for Zoology, Functional Peptidomics, University of Cologne, Cologne, Germany; 3 The Swire Institute of Marine Science and the School of Biological Sciences, The University of Hong Kong, Hong Kong, Hong Kong SAR; University of Padova, ITALY

## Abstract

The spatial distribution and the amount of intraspecific genetic variation of marine organisms are strongly influenced by many biotic and abiotic factors. Comparing biological and genetic data characterizing species living in the same habitat can help to elucidate the processes driving these variation patterns. Here, we present a comparative multispecies population genetic study on seven mangrove crabs co-occurring in the West Indian Ocean characterized by planktotrophic larvae with similar pelagic larval duration. Our main aim was to investigate whether a suite of biological, behavioural and ecological traits could affect genetic diversities of the study species in combination with historical demographic parameters. As possible current explanatory factors, we used the intertidal micro-habitat colonised by adult populations, various parameters of individual and population fecundity, and the timing of larval release. As the genetic marker, we used partial sequences of cytochrome oxidase subunit I gene. Genetic and ecological data were collected by the authors and/or gathered from primary literature. Permutational multiple regression models and ANOVA tests showed that species density and their reproductive output in combination with historical demographic parameters could explain the intraspecific genetic variation indexes across the seven species. In particular, species producing consistently less eggs per spawning event showed higher values of haplotype diversity. Moreover, Tajima’s *D* parameters well explained the recorded values for haplotype diversity and average *γ*st. We concluded that current intraspecific gene diversities in crabs inhabiting mangrove forests were affected by population fecundity as well as past demographic history. The results were also discussed in terms of management and conservation of fauna in the Western Indian Ocean mangroves.

## Introduction

The genetic structure of populations in the marine realm is a complex process affected by both biotic and abiotic factors including biological, ecological, and behavioural traits of adults and larval stages [1, 2]. Developmental mode is known to be one of the main features determining the dispersal ability of benthic marine invertebrates [3, 4]. A number of studies have shown that species with planktotrophic larvae have the potential to disperse further than species with lecithotrophic larvae or direct development [5–9]. Moreover, the existence of a positive correlation between pelagic larval duration (PLD) and gene flow is considered to be a general rule of marine organisms [10].

However, there are many striking exceptions to the above rule. Molecular tools have recently been used to show that the existence of a long pelagic larval phase is often not sufficient to result in genetically homogeneous populations [3, 4]. A great deal of variability in genetic diversity within and among marine populations may in fact arise due to both biotic and abiotic factors, acting as specific barriers that individuals cannot overcome [3, 4, 11–13]. In addition to historical barriers, existing abiotic barriers include local currents and gyres, which favour retention of larvae in near-shore areas, and variations in seawater temperature and salinity, which are deleterious for larval survival or development [14]. However, larval behavioural mechanisms such as vertical migrations [15] and life-history traits such as egg size and larval release sites [16] are biotic factors also playing important roles. Moreover, the unpredictability of the connectivity in marine ecosystems is enhanced by the fact that even species with similar life-history traits and/or living in the same geographical area can show contrasting patterns of genetic variation (for a review see [17]). Thus, how can we uncover the mechanisms underpinning the differences in intraspecific genetic variation patterns that may be observed in the marine realm?

A useful approach for identifying the historical and current factors that shape the spatial distribution and the degree of genetic variation may come from the comparison of closely related and sympatric species with similar biology sharing the same ecosystem [17, 18]. Defining the likely factors affecting genetic patterns on an ecosystem scale, such studies may also help in developing effective ecosystem-based management and in guiding the sustainable use of resources [18]. In fact, Kelly and Palumbi [18] used such an approach to compare the population genetic patterns of 50 marine invertebrate species along the Pacific coasts of North America. The authors showed that one of the most relevant structuring factors was the depth along the intertidal belt inhabited by the adults. García-Merchán et al. [19] showed that, among seven crustacean decapods present along the Atlantic-Mediterranean transition area, the shallow-water species exhibited higher gene diversities and stronger population genetic structures than the deep-water species.

We postulate the above approach could greatly help us to bridge the knowledge gap regarding the possible factors that shape the degree of intraspecific gene diversity and the population genetic structure in mangrove crabs. Mangroves are tropical and subtropical intertidal forests that colonise protected coastlines, such as creeks, lagoons, and estuaries [20]. They often present a fragmented distribution along the coastline. Mangroves are inhabited by specific invertebrate fauna with a high degree of specialization, that is radically different from those that colonise the stretches of coastline separating such forests [21]. In this ecosystem, crustacean decapods, mainly brachyuran crabs, are the most diverse and ecologically relevant taxa [21, 22]. In true mangrove crab families, females brood their embryos on their abdominal appendages for about 2 weeks [23, 24] before hatching the larvae out into the water column. This is followed by a planktotrophic larval phase and a final settlement stage called megalopa, where they ultimately settle in habitats typical of adult populations [25]. However, there are spatial and temporal differences in the reproductive patterns of mangrove crabs. Most of the species that belong to the Sesarmidae, Grapsidae, and Ocypodidae families show seasonal rhythms with clear synchronicity to the fortnightly and monthly cycles of tidal amplitude. In the majority of the studied species, spawning mostly occurred once a month during the highest spring tide of the synodic month, while in the other species the spawning occurred once a fortnight at each spring tide [23, 25–31]. The gecarcinid crabs, which occupy the supratidal area of the mangrove forests, have a seasonal and tidal cycle of larval release corresponding to the *en-masse* spawning peaks around the equinoctial spring tides [32]. In some portunid crabs, such as the commercially important species of the genus *Scylla*, ovigerous females migrate to the shelf where they release their larvae into open oceanic waters, thereby favouring larval export [33].

In this paper, we present a comparative multispecies study on the intraspecific gene diversities and population genetic structures of seven mangrove crabs co-occurring along the East African coast, characterized by similar PLD (approximately 1 month) but substantially different reproductive patterns. The main aim of this study was to investigate whether a suite of biological, behavioural, and ecological traits could affect the degree of genetic diversity within and among populations of the study species, ultimately affecting their demographic connectivity leading to different scenarios in terms of population genetic structures. As possible explanatory factors, we looked at the intertidal micro-habitat colonised by adult populations, and used various parameters of the individual and population fecundity and timing of the larval release. As a genetic marker, we sequenced the partial region of the cytochrome oxidase subunit I (COI). Genetic analyses were used to trace historical events in the West Indian Ocean that affected gene diversity and to unravel the conservation status of East African mangroves.

## Materials and Methods

### Study species and sample sites

**Fig 1:**
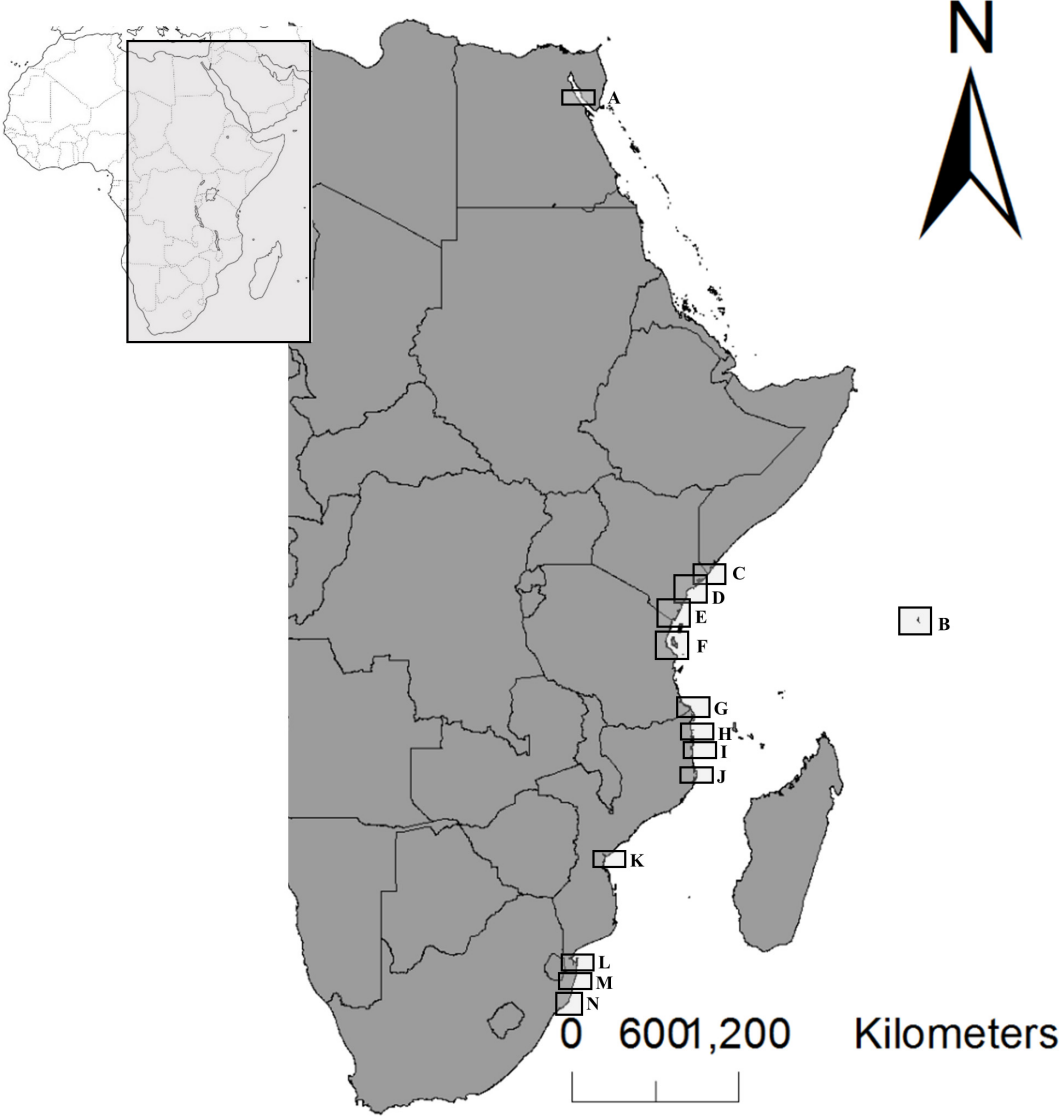
Sampling localities. Map of the West Indian Ocean with locations of population groups A-N. For each group, details of the populations sampled per each species are reported in S1 Table.

We selected seven mangrove crab species: *Uca inversa*, *Uca hesperiae*, *Uca occidentalis*, *Perisesarma guttatum*, *Neosarmatium africanum*, *Scylla serrata*, and *Cardisoma carnifex*. They belong to four different Brachyuran families and are all representative species of Western Indian Ocean mangrove fauna. Due to their distinct distribution patterns, not all of these species could be sampled at each location (Fig 1 and S1 Table). In particular, *U*. *inversa*, *N*. *africanum P*. *guttatum* are restricted to the east African coast and Madagascar Island [34–36], and thus they could not be collected in the Seychelles Islands (S1 Table). *U*. *hesperiae* and the newly described specie *U*. *occidentalis* (Naderloo, Schubart and Shih, 2016) are common along the whole western Indian Ocean [34, 37]; while the distribution ranges of *S*. *serrata* and *C*. *carnifex* extend to the whole Pacific region [38–40].

Genetic data for *U*. *inversa*, *U*. *hesperiae* and *C*. *carnifex* were obtained directly, which formed the original dataset of this paper (GenBank accession numbers: KX396518-34, KX396490-517 and KX396479-89, respectively). The genetic data of the other four species were totally or partially gathered from previous studies in the literature and supplemented with our collected data as detailed below and in S1 Table. We analyzed 120 individuals of *U*. *inversa* from six populations distributed from Egypt to Mozambique, 125 individuals of *U*. *hesperiae* from six populations distributed from Kenya to Mozambique, and 75 individuals of *C*. *carnifex* from five populations distributed from Kenya to Mozambique and Tanzania including Seychelles.

The population genetic structure of *U*. *occidentalis* (= *U*. *annulipes*) [37] was assessed by merging sequences analyzed by Silva and co-authors [41] with new sequences produced for this study from two Kenyan populations (Mida Creek and Lamu Island) and one population from Seychelles giving a total of 421 sequences (S1 Table) (GenBank accession numbers: KX396455-78). The samples collected by Silva et al. [41] came from 30 populations distributed along the East African coast covering Kenya, Tanzania, Mozambique, and the Republic of South Africa (S1 Table). With respect to our sampling design, data from single point locations [41] that were within a distance of approximately 50 km of one another within each geographical zone were merged to form 14 populations groups, A to N (S1 Table). This procedure allowed us to obtain a comparable sampling, as far as possible, of all the studied species.

The genetic dataset for *Perisesarma guttatum* included all the sequences published by Silva and co-workers [42] (GenBank accession numbers: KX374910-82), except those relating to populations collected in Southern Mozambique that were considered to be a cryptic species (S1 Table). The sampling of *P*. *guttatum* covered a geographical gradient from Kenya to Mozambique, including six study areas each with three to five single point locations at close range (i.e. about 50 Km). These populations were merged to form six groups in accordance with our sampling design (S1 Table).

Finally, the datasets for *S*. *serrata* [43, 44] and *N*. *africanum* [35, 45] remained unchanged with respect to the published datasets, and consisted of 190 sequences from seven locations distributed from Kenya to Mozambique and Seychelles Islands (GenBank accession numbers: AF279309-AF279332 and GUO55497-GUO55514), and 147 sequences from six locations exclusively sampled along the East African coast (GenBank accession numbers: FN392139-41, FN568275-97 and FN 568301–06), respectively.

### DNA extraction and amplification

For each specimen (i.e. adult individual), muscle tissue was removed from one pereiopod. The tissue was immediately placed in ethanol and the specimen was released into their natural environment. The genomic DNA was extracted using the Puregene Kit (Gentra System) and resuspended in TE buffer or distilled water and stored at -20°C.

A fragment of the cytochrome oxidase subunit I (COI), corresponding to the barcoding region and consisting of 656 base pairs (bp), was amplified using polymerase chain reaction (PCR) with the following primers: COL6b 5’-acaaatcataaagatatygg-3’ [46] and HCO2198 5’-taaacttcagggtgaccaaaaaatca-3’ [47]. The amplifications were performed in a Perkin Elmer 9600 thermal cycler with the following PCR conditions: 40 cycles of denaturation for 45 s at 94°C, annealing for 1 min at 48°C, extension for 1 min at 72°C, preceded by an initial denaturation for 10 min at 94°C followed by a final extension for 10 min at 72°C. Subsequently, PCR products were visualized on an agarose gel, purified by precipitation with *Sure Clean* (Bioline) and then resuspended in water. The sequence reactions were performed with the Big Dye terminator mix (Big Dye Terminator® V 1.1 Cycle Sequencing kit; Applied Biosystems) followed by electrophoresis in an ABI Prism automated sequencer (ABI Prism™ 310 Genetic Analyzer; Applied Biosystems). The sequences were corrected manually with Flinch TV 1.4.0 (Geospiza®) and aligned by eye with Bioedit version 7.2.5 [48]. Due to the high number of samples processed, only questionable haplotypes (for example sequences with overlapping peaks) were sequenced in both directions.

### Genetic data analysis

Genetic heterogeneity within each population and in the overall population was estimated for each species. The haplotype diversity (*h*), which indicates the probability that two randomly chosen haplotypes are different in a population [49], and the current nucleotide diversity (*θ*_π_), which indicates the percentage mean number of differences between all pairs of haplotypes in a population [49], were determined by ARLEQUIN version 3.5.2.2 [50]. We also calculated the historical genetic diversity (*θ*_w:_) [51] based on the number of segregating sites among the sequences using DnaSP version 5.1 [52]. The comparison between current and historical nucleotide diversities can provide information about the recent evolutionary history of the population, with *θ*_π_ > *θ*_w_ indicating a recent population growth and *θ*_π_ < *θ*_w_ indicating a population bottleneck [53].

A minimum spanning network was built for each species using NETWORK version 4.5.0.1 (Shareware Phylogenetic Network Software; http://www.fluxus-engineering.com/sharenet.htm) to investigate the geographic distribution of haplotypes.

For each species, the genetic differentiation among sampling sites was determined based on analysis of molecular variance (AMOVA) [54] as implemented in ARLEQUIN. Fixation indexes [55] were computed using haplotypic frequencies only, as well as using genetic distances. We used the Tajima and Nei model [56] as a distance method for unequal nucleotide frequencies, which are commonly reported in mtDNA of arthropod species [57]. Significance of the fixation indexes, under the null hypothesis of no differences among populations, was tested using a non-parametric permutation approach (10,000 permutations of haplotypes among populations). In this case, the P-value of the test was the proportion of permutations with fixation index values larger or equal to the observed one [54].

For each species we also calculated the Gammast (*γ*st) [58] as proxy of population subdivision. *γ*st values were calculated using DnaSP and their significance was tested using Snn statistics [59].

The spatial distribution of haplotypes among populations was also assessed by applying Exact tests [60] as implemented in ARLEQUIN using 10,000 Markov chain steps, and by contingency *χ*^*2*^ tables, without pooling rare haplotypes, using a Monte Carlo simulation [61] as implemented in CHIRXC [62]. Probability of heterogeneity in the haplotype distribution was estimated by comparing between observed and simulated *χ*^*2*^ values obtained from 20,000 random permutations of the original data.

The historical demographic history of our species was reconstructed applying the mismatch distribution analysis (i.e., the distribution of the observed number of differences between pairs of haplotypes) as implemented in ARLEQUIN. Populations at the equilibrium are expected to show a multimodal distribution of haplotype frequencies, while populations having recently passed through a demographic expansion or a range expansion are predicted to have a unimodal distribution [63]. From mismatch distribution of each species, we also calculated the raggedness index, *rg* [64, 65], which measures the smoothness of the mismatch distribution. The *rg* significance was tested by a parametric bootstrap approach (10,000 replicates) under the null hypothesis of population expansion as implement in ARLEQUIN.

We also estimated the expansion parameters Tau (*τ*) under both a demographic or spatial expansion hypothesis by using a generalized non-linear least squares approach [66]. The time (t) at which the demographic or spatial expansion began was calculated by applying Li’s formula [67], t = *τ*/2μ, where μ is the mutation rate per site per year. In our case μ is assumed to be 1.15% per million years for the portunid species *S*. *serrata* as calculated by [68], and 1.66% per million years for all other species as calculated for sesarmids from Jamaica by [69]. Approximate confidence intervals for the demographic parameters were obtained by 1,000 parametric bootstrap replicates.

In addition, we applied three neutrality tests based on different assumptions, Tajima’s *D* test [70], Fu’s *F*s test [71] and R2 tests [72]. Fu’s *F*s test uses information from the haplotype distribution, whereas Tajima’s *D* test and R2 test use information on the mutation frequency for assessing population expansion [72]. The significance levels of Tajima’s *D* test and Fu’s *F*s test were estimated by generating random samples under the null hypothesis of selective neutrality and population equilibrium using a coalescent simulation [73] as implemented in ARLEQUIN. The significance level of the R2 test was estimated using DnaSP based on 1,000 simulated re-sampling replicates.

### Biological, ecological, and reproductive traits

For each species, information on taxonomic family, distribution range, preferred mangrove habitat, number of spawning events per year, zone of spawning, and PLD were gathered from the literature. The average density of various East African populations of the selected species was also collected from various references, thus to calculate the average number of eggs produced by each population per unit square. The average number of eggs carried by females of *S*. *serrata* and *C*. *carnifex* were determined from literature reviews, while those of *U*. *inversa*, *U*. *occidentalis*, *U*. *hesperiae*, *P*. *guttatum* and *N*. *africanum* were estimated in the present study following the standard methodology for counting decapod eggs, recently implemented by Penha-Lopes et al. [74]. We collected 150 ovigerous females from each of *U*. *inversa*, *U*. *occidentalis*, *U*. *hesperiae*, and *P*. *guttatum*, and 65 of *N*. *africanum* at various sites along the Kenyan coast, both in dry and wet seasons between 2008 and 2009. For each study species, 50 eggs were collected from each of 10 randomly chosen females and placed in petri dishes with seawater. The diameter of each egg was immediately measured to the nearest 0.01 mm under a microscope with a calibrated micrometer eyepiece. Egg volume (V, mm^3^) was calculated as the volume of a sphere. The eggs were then separated from the pleopods with diluted bleach. They were placed between two transparent sheets and photographed using a Canon EOS digital camera at a distance of 40 cm with a 50 mm 2.8 lens. Egg counting at each embryonic stage in each population for each species was carried out using Image J software after adjustments and calibrations. An error estimation was performed by manually counting the eggs in 10% of all the photographs and comparing with the counts given by the software. A subsample of 50 specimens of each of the tested species was randomly selected to record their carapace width (CW) using Vernier calipers. In addition, CW measurements were performed in 20 specimens of *C*. *carnifex* and 50 of *S*. *serrata*.

On the basis of the above data on the number of spawning event per year, the average number of eggs carried per female and their average density per m^2^, we calculated the average amount of eggs per m^2^ for each population, the amount of eggs produced per year by each female, and the amount of eggs produced per year per m^2^. The above estimates per surface area were corrected by a factor based on the average percentage coverage of the vegetation belts particular to each species in the East African mangrove forests (S2 Table).

### Comparison between biological characteristics and genetic variables

We tested for significant correlations between population genetic variables and a set of twelve biological and one genetic characters. The Tajima’s *D* parameter, as suggested by Kelly and Palumbi [73], and a set of biological characters (see S2 Table), either numerical (e.g., the amount of eggs produced per year by each female) or categorical (e.g., distribution range), were entered as independent variables in permutational multiple linear regression and ANOVA models, respectively, with genetic parameters (*γ*st and haplotype diversity) used as the numerical dependent variables. Average *γ*st values were chosen as a proxy of population subdivision, since they give a more reliable interpretation of genetic differentiation. This parameter actually represents an unbiased estimate of *F*st that corrects for errors associated with incomplete sampling of populations and is more suitable for mitochondrial haplotype data [59]. Analyses involving haplotype diversity as dependent variable were also run excluding *U*. *occidentalis*, for an in-depth discussion about the biotic causes of such diversity (for more explanations see the Results and Discussion sections).

Prior to the analyses, we checked for multicollinearity among independent variables using draftsman plots and for heteroscedasticity of the dependent variables by applying Cochran’s test, and then consequently log-transformed the haplotype diversity parameters. All analyses were performed using PRIMER v. 6.1 [75] with the PERMANOVA+ for PRIMER routines [76].

### Ethical Statement

None of the sampled species, for both genetic and reproductive traits analyses, are endangered or protected by any international of national legal frame. The sampling of specimens was carried out under the frame of the PUMPSEA project (INCO-CT2004-510863), which held a Research Permit issued by the Kenya Marine and Fisheries Research Institute (KMFRI).

## Results

### Genetic diversity indexes

**Fig 2:**
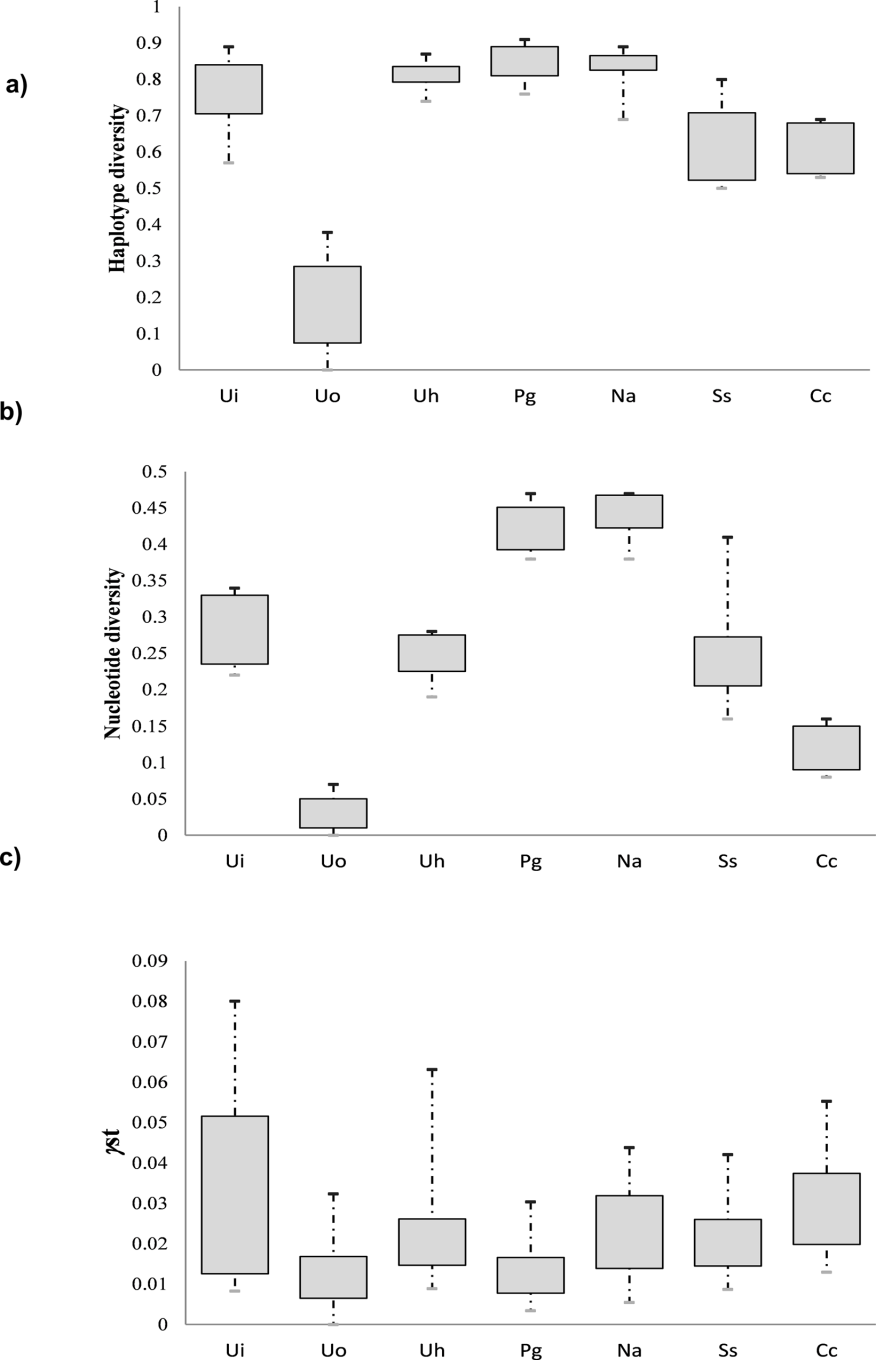
Genetic indexes in the seven study species. Boxplots for the haplotype diversity (a), nucleotide diversity (b), and *γ*st (c) in the seven mangrove crab species. The first and third quartiles were used to construct the box. Ui: *Uca inversa*; Uo: *Uca occidentalis*; Uh: *Uca hesperiae*; Pg: *Perisesarma guttatum*; Na: *Neosarmatium africanum*; Ss: *Scylla serrata*; Cc: *Cardisoma carnifex*.

We analyzed a fragment of the mtDNA gene encoding for the COI consisting of 550 to 650 bp for all study species. The haplotype diversity index was found to be high in all species (range 0.62 to 0.85), except in *U*. *occidentalis* that only had 18 haplotypes recorded in more than 400 individuals (Table 1; Fig 2). The highest haplotype diversity values were in the two sesarmid species, *P*. *guttatum* and *N*. *africanum*. However, the current nucleotide diversities *θ*_π_ were generally low and did not reach higher than 0.5% (Table 1; Fig 2). The value recorded for *U*. *occidentalis* was particularly low (0.03%) since all haplotypes only differed by very few mutations (Table 1; Fig 2).

**Table 1:** Diversity indexes within the seven mangrove crab species.

Species	N	Nhap	Vs	*h*	*θ*_π_	*γ*st
*U*. *inversa*	120	26	18	0.78 +/- 0.03	0.29 +/- 0.19	0.056
*U*. *occidentalis*	421	18	17	0.19 +/- 0.03	0.03 +/- 0.04	0.029
*U*. *hesperiae*	125	28	23	0.80 +/- 0.02	0.25+/- 0.16	0.040
*P*. *guttatum*	224	60	47	0.85 +/- 0.02	0.42 +/- 0.25	0.019
*N*. *africanum*	147	32	31	0.82 +/- 0.02	0.46 +/- 0.26	**0.039**
*S*. *serrata*	190	42	45	0.62 +/- 0.40	0.26 +/- 0.18	**0.034**
*C*. *carnifex*	78	11	9	0.63 +/- 0.03	0.12 +/- 0.10	0.046

Values shown are N: number of specimens analyzed, Nhap: number of haplotypes, Vs: number of segregating sites, *h*: haplotype diversity, *θ*_π_: nucleotide diversity (expressed in percentage), and average pairwise *γ*st values (significant values are shown in bold).

Current and historical genetic diversity values for each population group of the seven studied mangrove crabs are shown in Table 2. For all the species, all or most of the populations had historical nucleotide diversity values higher than current nucleotide diversity values, which clearly demonstrates a signature of recent population bottleneck as expected for populations that have been heavily exploited in the recent past.

**Table 2:** Nucleotide diversity for population groups.

Group	*U*. *inversa*		*U*. *occidentalis*		*U*. *hesperiae*		*P*. *guttatum*		*N*. *africanum*		*S*. *serrata*		*C*. *carnifex*	
	*θ*_π_	*θ*_w_	*θ*_π_	*θ*_w_	*θ*_π_	*θ*_w_	*θ*_π_	*θ*_w_	*θ*_π_	*θ*_w_	*θ*_π_	*θ*_w_	*θ*_π_	*θ*_w_
**A**	0.28	0.36												
**B**			0.00	0.00	0.24	0.31					0.16	0.24	0.16	0.19
**C**	0.22	0.27	0.00	0.00	0.19	0.22			0.43	0.47	0.22	0.71	0.15	0.19
**D**	0.34	0.32	0.00	0.00	0.28	0.28			0.47	0.46	0.41	0.99	0.08	0.05
**E**	0.30	0.3	0.03	0.04	0.28	0.39	0.38	0.51	0.42	0.50	0.28	0.66	0.09	0.09
**F**	0.34	0.45	0.06	0.16	0.22	0.26	0.47	0.77	0.38	0.34	0.25	0.59	0.14	0.19
**G**			0.07	0.20			0.46	0.77						
**H**			0.01	0.03			0.43	0.57						
**I**			0.01	0.07			0.43	0.76						
**J**			0.02	0.19			0.38	0.35						
**K**			0.03	0.09										
**L**	0.22	0.27	0.05	0.11	0.26	0.39			0.46	0.46	0.20	0.43		
**M**			0.03	0.08					0.47	0.53				
**N**			0.05	0.14										
**TOT**	0.29	0.54	0.03	0.41	0.25	0.68	0.42	1.21	0.46	0.9	0.26	1.40	0.12	0.29

Values shown are current (*θ*_π_) and historical (*θ*_w_) nucleotide diversity (expressed in percentage). Population groups correspond to those reported in S1 Table.

### Population genetic structures

The AMOVA tests revealed a lack of partitioning of the genetic variation in all the studied species (Table 3). The pairwise *F*st/ *Φ*st comparison results showed no significant differences for *U*. *hesperiae*, *P*. *guttatum*, *N*. *africanum*, *S*. *serrata*, and *C*. *carnifex*. For *U*. *inversa*, the pairwise population comparison results showed significant differences between Mida and Lamu and between Lamu and Ras Dege based on haplotype and nucleotide diversity indexes, as well as between Mida and Inhaca based on only nucleotide diversity values (data not shown). For *U*. *occidentalis*, we found the populations of Punta Rosa had differences compared to most other populations sampled by Silva et al. [41] and the three populations (Mida, Lamu, and Mahé Island) specifically analyzed in this study (data not shown).

**Table 3:** Analysis of Molecular Variance (AMOVA).

AMOVA	Source of variation	SS	Var	*Φ*st*/F*st	P
***U*. *inversa***				* *	
**Haplotype diversity**	Among population	2.57	1.66	0.016	0.10
	Within population	43.9	98.34		
**Genetic diversity**	Among population	6.01	1.74	0.017	0.15
	Within population	101.25	98.26		
***U*. *occidentalis***					
**Haplotype diversity**	Among population	1.37	0.74	0.009	0.21
	Within population	37.91	99.26		
**Genetic diversity**	Among population	1.21	0.11	0.001	0.41
	Within population	39.64	99.89		
***U*. *hesperiae***					
**Haplotype diversity**	Among population	1.89	-0.33	-0.003	0.53
	Within population	48.2	100.33		
**Genetic diversity**	Among population	3.92	-0.01	-0.0001	0.43
	Within population	93.74	100.01		
***P*. *guttatum***					
**Haplotype diversity**	Among population	2.03	-0.11	-0.001	0.51
	Within population	92.43	100.11		
**Genetic diversity**	Among population	5.81	-0.46	-0.004	0.65
	Within population	305.98	100.46		
***N*. *africanum***					
**Haplotype diversity**	Among population	2.38	0.64	0.006	0.24
	Within population	58.17	99.36		
**Genetic diversity**	Among population	7.79	0.54	0.005	0.30
	Within population	194.12	99.46		
***S*. *serrata***					
**Haplotype diversity**	Among population	2.1	0.48	0.004	0.26
	Within population	56.48	99.52		
**Genetic diversity**	Among population	4.4	0.24	0.002	0.34
	Within population	126.23	99.76		
***C*. *carnifex***					
**Haplotype diversity**	Among population	1.58	1.71	0.017	0.25
	Within population	22.68	98.29		
**Genetic diversity**	Among population	1.39	-0.77	-0.008	0.51
	Within population	28.77	100.77		

Results of partitioning genetic variation among populations for each study species, based on haplotype frequencies and on genetic diversity data [51]. SS: Sum of squares, Var: percentage of total variation, *Φst/F*st: F-statistics, P: P-values.

Conversely, the other statistical tests applied to analyze global genetic differentiation among populations (i.e. *γ*st, Exact test of population differentiation and contingency *χ*^*2*^ tables of haplotype frequencies) revealed the occurrence of population structures for some species. This was recorded for *N*. *africanum* and *S*. *serrata* based on *γ*st values (Table 1); for *N*. *africanum* (P = 0.05; significant pairwise comparison: Durban vs. Dar Er Saalam) and *U*. *inversa* (P = 0.05; significant pairwise comparisons: Lamu vs. Mida, Lamu vs. Dar Er Saalam, Shark’s Bay vs. Mida, and Shark’s Bay vs. Dar Er Saalam) based on the Exact test of population differentiation; and for *S*. *serrata* (*χ*^*2*^ = 265.5, df = 246, P = 0.04) from the contingency *χ*^*2*^ tables of haplotype frequencies.

**Fig 3:**
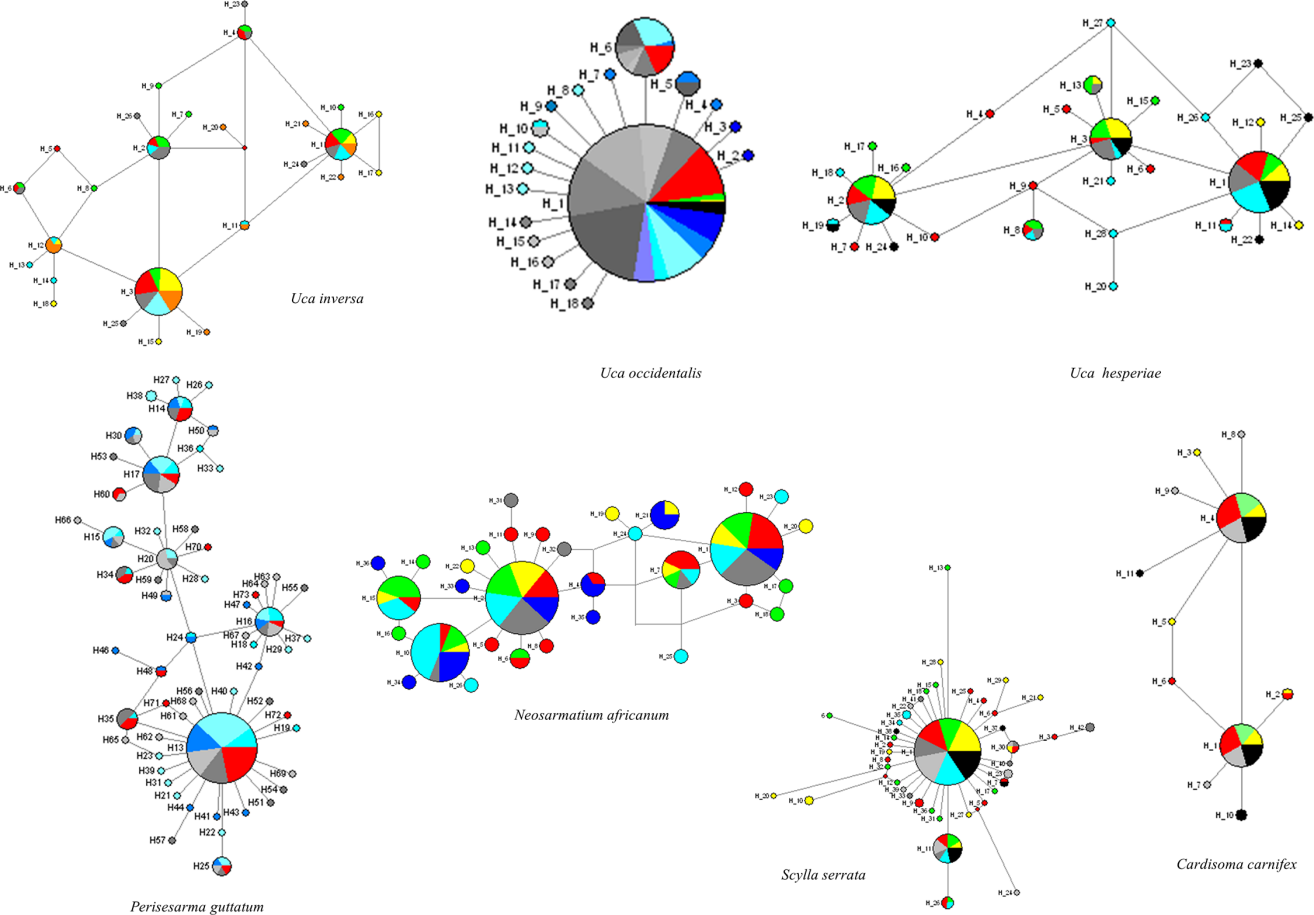
Median-joining haplotype networks. Minimum spanning trees showing the relationships among haplotypes of mitochondrial DNA COI for the seven study species. Each line length is proportional to the numbers of mutational steps. Circles representing haplotypes are scaled to their frequencies. Different colours indicate different populations.

The network of phylogeographic relationships among all haplotypes was reconstructed for each species (Fig 3). *U*. *inversa*, *U*. *occidentalis*, *S*. *serrata* and *C*. *carnifex* had star-like shaped networks with one or two unique haplotypes common to most of individuals distributed in all populations, and several derived haplotypes only found in one population. Most of the private haplotypes were singleton (present in one individual only) and were differentiated from the more widespread haplotypes by very few mutational steps. The networks of the other three species (*P*. *guttatum*, *N*. *africanum* and *U*. *hesperiae*) were more complex and articulated, especially those of the two sesarmid species that in fact had the highest genetic diversity values; however, no evident association between haplotypes and geography occurred in these species too.

### Demographic history analysis

**Fig 4:**
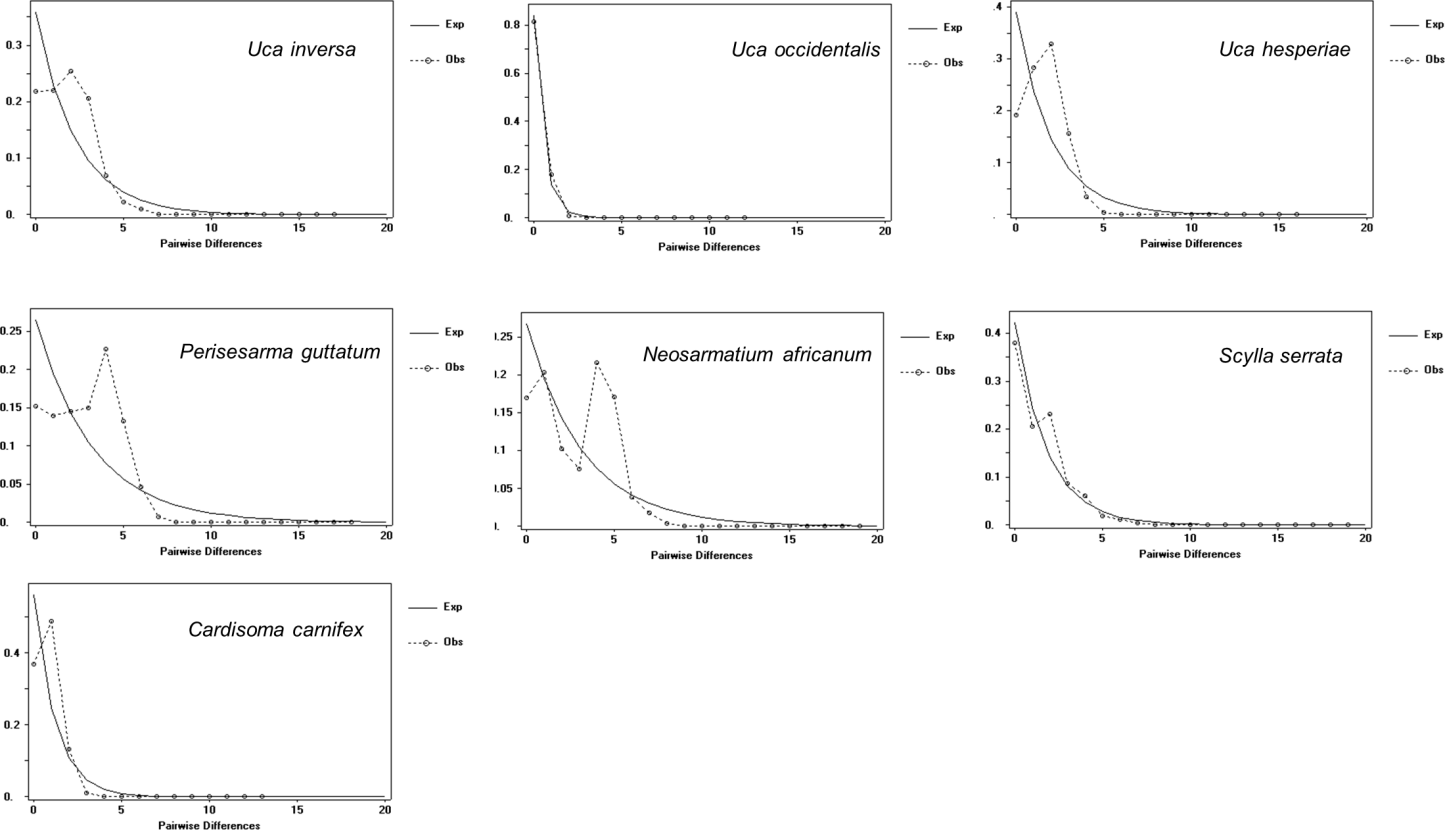
Mismatch distribution patterns. Mismatch distributions for the whole population of each of the seven study species. Observed (dotted line) and expected (continuous line) distribution under a sudden demographic expansion.

The mismatch distributions calculated for the whole population in each of the seven species had a unimodal distribution (Fig 4), as would be expected for populations that have recently experienced a demographic or range expansion [63, 77, 78].

At the same time, based on the raggedness index, *rg*, it was not possible to reject the null hypothesis of populations being out of equilibrium in any of the studied species except for *C*. *carnifex* (Table 4). Tajima’s *D* test, Fu’s *F*s test and R2 test also recorded a significant deviation from the equilibrium for all the species, with the exception of Tajima’s D test for *U*. *inversa* and *C*. *carnifex* (Table 4).

**Table 4:** Demographic analyses.

Species	T-D	F-Fs	R2	rg	Tau 1	Tau 2	t1	t2
*U*. *inversa*	-1.3	**-20.03**	**0.16**	0.02	2.31 (0.62–3.96)	2.11 (0.63–3.29)	1.79	1.62
*U*. *occidentalis*	**-2.23**	**-33.96**	**0.16**	0.2	3.0 (0.40–3.0)	0.08 (0.06–0.87)	2.02	0.42
*U*. *hesperiae*	**-1.82**	**-25.87**	**0.15**	0.05	1.72 (1.43–2.10)	1.72 (1.02–2.12)	1.44	1.36
*P*. *guttatum*	**-1.89**	**-26.36**	**0.16**	0.02	3.95 (1.45–6.45)	3.15 (1.19–5.04)	3.10	2.73
*N*. *africanum*	**-1.48**	**-19.82**	**0.04**	0.05	4.59 (1.15–8.11)	2.53 (0.71–6.39)	3.54	3.31
*S*. *serrata*	**-2.43**	**-28.69**	**0.16**	0.05	2.67 (0.02–5.03)	1.81 (0.43–3.62)	1.49	1.10
*C*. *carnifex*	-1.48	**-7.32**	**0.16**	**0.16**	0.93 (0.64–1.38)	0.93 (0.48–1.28)	0.78	0.74

Values shown are T-D: Tajima’s *D* test, F-Fs: Fu’s *F*s test, R2: Ramos-Onsis and Rozas’ R2, *r*g: raggedness index for the mismatch distribution, Tau 1 and Tau 2 are Tau mean values under sudden demographic and spatial expansion models with 95% confidence interval in brackets, t1 and t2 are time values in millions of years under sudden demographic and spatial expansion models. The significant P values are reported in bold.

Applying Li’s formula [67], t = *τ*/2μ, using the mean values of Tau with the assumption that a population had suddenly passed through a demographic expansion, we calculated the time at which each species began its population expansion (Table 4). These times were rather different among the seven species. The two sesarmid species seemed to have begun their current demographic expansion over 3 million years ago (Mya), the three ocypodid crabs and the portunid crab started around 1.5 to 2 Mya, whereas the results for *C*. *carnifex* indicated its population demographic expansion began less than 1 Mya. When a scenario of spatial expansion was considered, the values of Tau did not vary or were slightly decreased for *U*. *inversa*, *U hesperiae* and *C*. *carnifex*. *P*. *guttatum*, *N*. *africanum*, and *S*. *serrata*, indicating a higher decrease in their Tau values and thus the corresponding expansion times were lower even for the same temporal order of magnitude (Table 4). *U*. *occidentalis*, instead, showed a consistent decrease in its Tau value under a scenario of spatially expanding population, corresponding to an expansion time of 0.07 Mya.

### Biological traits and their correlations with genetic indexes

A list of primary ecological and reproductive traits of the study species is shown in Table 5, while in S2 Table this list is supplemented with the additional derived biological parameters used as independent variables in the statistical analyses. The studied mangrove crabs greatly differed in their population density and reproductive output, with the average number of eggs produced per female per spawning event ranging from 1,600 to 2,000,000. This fecundity parameter when combined with the differences in the population density and in the number of spawning events per year resulted in huge differences in the total estimated number of eggs produced by the various populations per unit area at each spawning event (e.g., the parameter eggs m^-2^ corrected, ranging from about 1,850 to 36,000, for *U*. *hesperiae* and *S*. *serrata*, respectively; S2 Table) and in the amount of eggs produced per year by the populations themselves (e.g. the parameter eggs m^-2^ year^-1^ corrected, ranging from about 22,400 to about 432,000 for *U*. *hesperiae* and *S*. *serrata*, respectively). Also within the genus *Uca*, we found consistent differences in populations of *U*. *hesperiae*, *U*. *inversa*, and *U*. *occidentalis* who produced about 22,400, 28,000 and 81,648 eggs m^-2^ year^-1^, respectively. Nevertheless, the PLD of these species was roughly the same (S2 Table).

**Table 5:** Main life-history and ecological traits of the seven mangrove crab species.

Species	*U*. *inversa*	*U*. *occidentalis*	*U*. *hesperiae*	*P*. *guttatum*	*N*. *africanum*	*S*. *serrata*	*C*. *carnifex*	References
**Family**	Ocypodidae	Ocypodidae	Ocypodidae	Sesarmidae	Sesarmidae	Portunidae	Gecarcinidae	
**Distribution range**	EAM	WIO	WIO	EAM	EAM	IPO	IPO	[34–40]
**Mangrove habitat**	Littoral fringe	Littoral fringe	Sublittoral fringe	Eulittoral	Littoral fringe	Eulittoral/sublittoral fringe	Supralittoral	[34, 79–82]
**Spawning events year**^**-1**^	10	12	12	12	4	12	2	[23, 83–85]
**Zone of spawning**	Littoral fringe	Littoral fringe	Sublittoral fringe	Eulittoral	Littoral fringe	Oceanic platform	Sublittoral fringe	[23, 33, 83]
**PLD (days)**	26	28	26	23	29	26	25	[41, 86–91]
**Av. density (ind m**^**-2**^**)**	7.77	12.6	4.75	1.4	0.59	0.08	0.725	[82, 92, 93]
**Max CW(mm)**	20 *	20 *	20 *	20 *	20 *	20 *	20 *	/
**Egg female**^**-1**^ **spawning**^**-1**^	1600*	2400*	1750*	8200*	56700*	2000000	695000*	[85, 94, 95]

Data shown are Family; Distribution range (EAM: East Africa and Madagascar; WIO: West Indian Ocean, IPO: Indo-Pacific Ocean); Mangrove habitat occupied by adult populations; Number of spawning events per year; Zone of spawning; PLD: pelagic larval duration; Av. density: average density of adult populations; Max CW: Maximum adult carapace weight; Egg female^-1^ spawning^-1^: average number of eggs produced per female per spawning event. Data collected and calculated from the authors for this paper are indicated with an asterisk.

**Fig 5:**
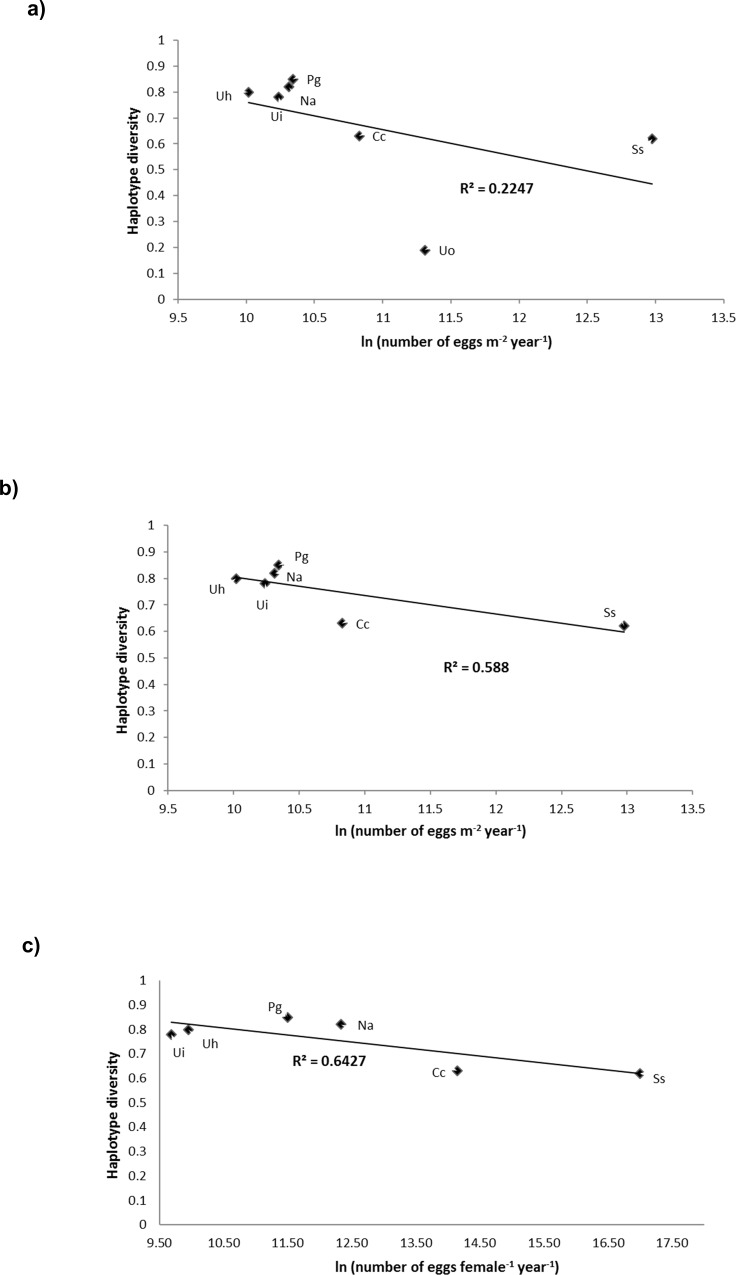
Correlates of intraspecific genetic diversity indexes. Correlation between the haplotype diversity and number of eggs/m^2^ per year including (a) and excluding (b) *U*. *occidentalis;* and haplotypes diversity and number of eggs/female per year excluding *U*. *occidentalis* (c). Ui: *Uca inversa*; Uo: *Uca occidentalis*; Uh: *Uca hesperiae*; Pg: *Perisesarma guttatum*; Na: *Neosarmatium africanum*; Ss: *Scylla serrata*; Cc: *Cardisoma carnifex*.

**Fig 6:**
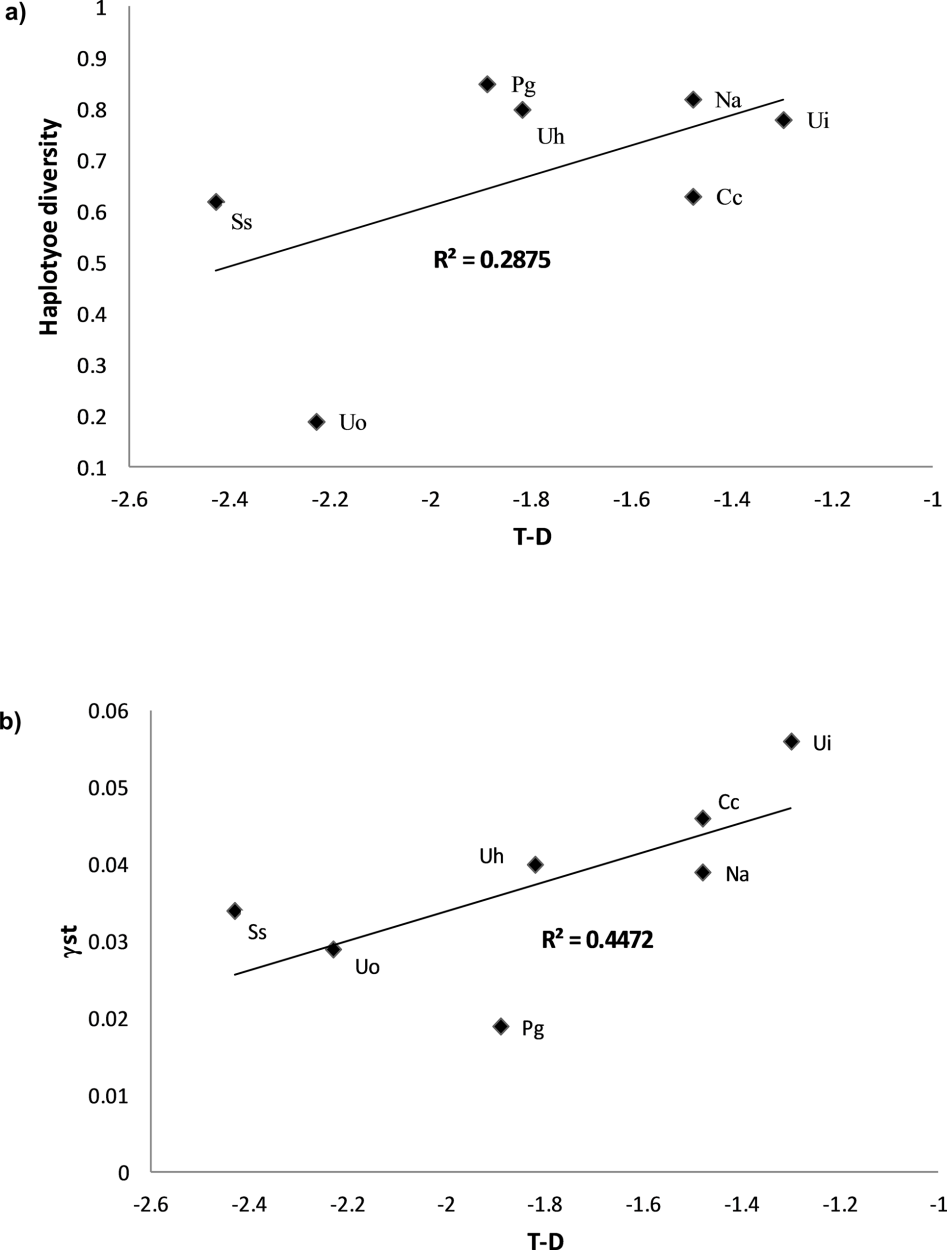
Correlates of intraspecific genetic diversity indexes. Correlation between the haplotype diversity and Tajima’s D parameters (a); and the *γ*st values and Tajima’s D parameters (b). Ui: *Uca inversa*; Uo: *Uca occidentalis*; Uh: *Uca hesperiae*; Pg: *Perisesarma guttatum*; Na: *Neosarmatium africanum*; Ss: *Scylla serrata*; Cc: *Cardisoma carnifex*.

It is interesting to underline that reproductive output, expressed as amount of eggs produced per female per spawning event, is strongly and positively correlated to adult dimensions (R^2^ = 0.89, p = 0.001; Linear regression test) confirming that as big a crab species is as it produces more eggs [96]. Permutational multiple regression models showed that the estimated number of eggs m^-2^ at each spawning event was negatively correlated with the haplotype diversity, while the species average density and Tajima’s D parameter were positively correlated to haplotype diversity. Overall, these variables accounted for 99% of its variability (R^2^ = 0.998, AIC criterion, permutational multiple regression test, Figs 5A and 6A, S3 Table). This analysis was also run excluding *U*. *occidentalis* due to its unexpected low value of haplotype diversity. The results of these new analyses clearly confirmed how the reproductive parameters (amount of eggs m^-2^ produced at each spawning event, as well as every year) are strongly, and inversely, related to haplotype diversity, accounting for 99% of its variability, (R^2^ = 0.99, AIC criterion, permutational multiple regression test, Fig 5B, S3 Table).

ANOVA tests did not show any significant influence of the geographical range, taxonomic families, temporal and spatial spawning patterns, and intertidal level occupied by adult populations on the selected parameter.

The same statistical approach showed that both biological traits and Tajima’s D parameter correlated with *γ*st. The model considering the average density, total amount of eggs produced per female per year and Tajima’s *D* as the descriptors could explain about 98% of the total variability in terms of *γ*st (R^2^ = 0.979, AIC criterion, permutational multiple regression test, S3 Table), with the latter descriptor being the best correlate (Fig 6B). Again, ANOVA tests did not show any significant influence of geographical range, taxonomic families, temporal and spatial spawning patterns, and intertidal level occupied by adult populations.

## Discussion

A complex combination of historical and current biotic and abiotic factors can shape the patterns of genetic variation in natural populations [1, 2], but it is often very difficult to disentangle the relative contributions of these factors, especially when studying one or only a few species. Conversely, multispecies comparative studies, such as this study, can provide insights that cannot be derived from single species analyses [17]. Our study is novel in this field of research because we showed that the reproductive output of seven mangrove crabs together with their demographic history strongly influenced their gene variation and population genetic structure, regardless of their PLD. Although a large number of genetic studies have examined the influence of developmental mode and PLD on genetic diversity and connectivity of marine species, only a few have investigated the effects of reproductive output [97] and other ecological and behavioral traits on gene variation [18, 19]. Indeed, our findings confirmed that unraveling the causes of intraspecific patterns of genetic variation in marine species is a complex task, since current and historical factors act on different time scales but in strict synergy.

The target species in the present study are representative components of mangrove fauna in the Western Indian Ocean as they are abundant in most mangrove swamps in this region [81, 92]. All seven species are broadcast spawners that disperse planktotrophic larvae with similar PLDs (around 3–4 weeks), but they showed substantial differences in terms of their reproductive output. These differences can be ascribed to variations in the population density, rhythm of their spawning patterns and fertility, which had direct and strong consequences on the genetic dissimilarities shown in the studied species. Our results showed the studied species could be roughly split into three groups, irrespective of their phylogenetic relationships. The first group included *U*. *hesperiae*, *U*. *inversa*, *P*. *guttatum* and *N*. *africanum* which produced 20,000–30,000 eggs m^-2^ year^-1^. The second group included *C*. *carnifex* and *U*. *occidentalis* which produced 50,000–80,000 eggs m^-2^ year^-1^. Finally, the third group included *S*. *serrata* which produced up to 430,000 eggs m^-2^ year^-1^. Based on haplotype diversity, the first group had a high diversity (about 0.8 or higher), while the species of the other two groups had intermediate haplotype diversity values (about 0.6, *S*. *serrata* and *C*. *carnifex*) or very low diversity (i.e *U*. *occidentalis*). Although less prominent than the haplotype diversity, differences in the nucleotide diversity were also observed, but these only closely matched the above groupings based on reproductive outputs and haplotype diversity. The two sesarmid species, *P*. *guttatum* and *N*. *africanum*, had the highest nucleotide diversity (about 0.5%); *U*. *inversa*, *U*. *hesperiae*, *S*. *serrata*, and *C*. *carnifex* had intermediate diversity (about 0.2%); and *U*. *occidentalis* had impressively low diversity (0.03%), which was an order of magnitude lower than the other species.

From a reproductive point of view, the impressive egg production capacity of *S*. *serrata* was far greater than that of the other species, which could be explained by their physical dimensions and high quality diet. In fact, *S*. *serrata* are the largest mangrove crab species in the Indian Ocean and are the top predator feeding on molluscs, crabs, and fish [98, 99]. In contrast, *C*. *carnifex*, *P*. *guttatum*, and *N*. *africanum* are mainly herbivores [82, 100] and the three *Uca* species are filter feeders who eat microalgae and bacteria [93, 101]. Similar to *S*. *serrata*, the dimension of *C*. *carnifex* could explain their high reproductive output, as they have large adult dimensions at around five times the size of the other species. *U*. *occidentalis*, the smallest crab species in the study, displayed a combination of high reproductive output (about a third more than the other *Uca* species) and high population densities (about two to 20 times more than the other *Uca* species and Sesarmidae), which could explain its large annual egg production per unit area.

Regarding population genetic structure, high levels of gene flow were recorded for all the species. However, here was some evidence of weak population structures that emerged for *N*. *africanum*, *S*. *serrata*, *U*. *inversa* and *U*. *occidentalis* based on the population pairwise *F*st comparisons, *γ*st, *χ*^*2*^ tables, or Exact test results. These results might indicate the existence of differential dispersal abilities among the seven species, which was also suggested by the diverse shapes of the haplotype networks. However, such differences could have been masked by recent historical demographic events and exchange of individuals based on a stepping stone population model [102].

All the species showed clear signs of recent population bottlenecks or selection events, as demonstrated by mismatch distribution patterns and the significant values from Tajima’s *D*, Fu’s *F*s and R2 tests. Based on the demographic parameters of the mismatch distribution analysis under a demographic or spatial expansion model, we calculated the times at which Western Indian Ocean metapopulations of these species could have expanded. These historical events appeared to have occurred at different times for these diverse species, but all were during the Pleistocene period. This epoch could be significant because the Western Indian Ocean has been impacted by great climatic changes, primarily induced by the closing of the Indonesia Gateway around 3–4 Mya [103, 104] and by successive changes in the intensity and phases of the Indian Monsoon [105].

Within our studies species *U*. *occidentalis* was a peculiar case, because it had low haplotype and nucleotide diversity values rarely associated with broadcast spawners [106] and this could indicate strong bottlenecks due to founder effects, recent colonization of the studied area or directional selection [19, 107, 108]. On the other hand, high haplotype and moderate nucleotide values recorded for the other six species are in line with values reported for other crustacean species with planktonic larvae [106], and generally describe populations with large numbers of closely related haplotypes [109]. This could indicate the rapid growth of a population, after a decrease in its effective population size, enhancing the retention of new mutations [109]. This hypothesis is more than plausible for our study species, that experienced demographic or spatial expansions during Pleistocene period. During this era, sea temperature fluctuations and alternating glacial and interglacial periods could have affected dispersion, survival, and recruitment of planktonic larvae, altering the effective population sizes.

Overall, our data suggested that species characterized by lower reproductive output such as *U*. *hesperiae*, *U*. *inversa*, *P*. *guttatum* and *N*. *africanum*, exhibited higher haplotype diversity and *γ*st values. This correlation can be possibly explained by the hypothesis that broadcast spawners with similar PLD would have a higher chance of dispersing their larvae over larger distances to find suitable mangrove habitats if the numbers of produced larvae were higher. In fact, it is well known that dispersion and recruitment of mangrove crab larvae are related to stochastic events such as offshore wind stress and to deterministic effects such as tidal cycles [110, 111]. Thus, a high reproductive output can maximise their chances of achieving a particular combination of factors needed to successfully settle in irregularly distributed mangrove habitats such as in creeks and estuaries. We hypothesise that the correlation between haplotype diversity and reproductive output represents a genetic signature of sweepstake events, recognised as prominent features of recruitment in the sea [112]. Species such as mangrove crabs with planktonic larval stages are generally characterised by highly variable seasonal reproductive success due to stochastic events that influence larval mortality rates and recruitment success. This means at every recruitment event, only a small subset of larvae will contribute to the next generation of adults [112]. Hence, in species where a high number of females contribute to the larval pool with a low number of larvae per capita such as *U*. *hesperiae*, *U*. *inversa*, *P*. *guttatum* and *N*. *africanum*, the surviving subset should be generally quite heterogeneous, resulting in a constant increase of the haplotype diversity over time. On the other hand, when few females are contributing to large numbers of larvae per capita such as *S*. *serrata* and *C*. *carnifex*, at every spawning event the larval pool should be genetically homogenous, and should be independent from stochastic recruitment and even from the next generation of adults.

Finally, our findings indicated that distributional range, taxonomic families, temporal and spatial spawning patterns, and the intertidal level occupied by adult populations did not influence the genetic variability index and gene flow estimation. We cannot completely disregard the effects of these factors, which might not have been detected by our analyses due to the limited number of study species and the use of mtDNA alone. Over the past four decades, mtDNA has been the most common marker in population genetic and phylogeographic studies, mainly for its supposed low recombination and effective neutrality [3, 4]. Recently, much controversy has been generated because some studies using mtDNA could not confirm the above-mentioned assumptions [113, 114]. Moreover, some studies combining mtDNA and nuclear loci found conflicting results when comparing these two genetic markers, the so-called mito-nuclear discordance [115]. In our study, we can reasonably exclude some causes of the above mito-nuclear discordance, such as the sex-biased dispersal asymmetry [115], since it is highly unlikely that mtDNA-derived dispersal estimates may represent female dispersal alone in mangrove crabs characterized by sedentary adults that disperse through planktonic larvae [116]. Nevertheless, we are aware that our novel results of the influence of reproductive output on patterns of intraspecific genetic variation in mangrove crabs need to be complemented with further studies based on variable nuclear markers such as SSRs and SNPs.

## Conclusions

This study sheds new light on the population ecology and dynamics of mangrove crabs, and demonstrates how reproductive output is a crucial biological trait affecting genetic diversity and population structure, even in combination with signatures of recent past events. Apart from the ecological relevance, our findings are important for developing conservation strategies for the management of Western Indian Ocean mangroves [18]. Mangrove forests are disappearing worldwide at an impressive rate, mostly due to local mismanagement [117], which consequently poses a serious risk of the extinction of their unique fauna and flora. Our findings should be a further warning, as we demonstrated that all seven of the studied mangrove crab species bear clear marks of recent population bottlenecks (*θ*_*π*_< *θ*_w_). This is to be expected for populations recently overexploited and drastically reduced in size. *S*. *serrata* distributed throughout the East African coast is a commercially important species, which is consumed by the local population and sold to tourists, and are thus very likely to be overexploited [44]. On the other hand, the other study species are not exploited by local communities but also experienced recent reductions in population size, possibly due to mangrove area loss by direct destruction or indirect degradation by pollution. Experimental, ecological and behavioral evidence directly support the hypothesis that mangrove degradation primarily affects the macrofauna. In fact, East African crab and mollusc populations have shown signs of cryptic and subtle ecological and biological degradation in a number of mangrove sites, whereas the trees seemed to be relatively unaffected [74, 92, 93]. We believe the above genetic and ecological signals of degradation of the mangrove fauna can no longer be ignored and we urge the development of effective ecosystem-based management and guidelines for the sustainable use of these valuable ecosystems.

## Supporting Information

S1 Table Sampling locations. For each species, the number of sequences analyzed in each locality is reported. Population groups correspond to localities as used in the statistical analyses. Sequences were obtained for this paper and from literature (see details in the text).
(DOCX)

S2 Table Data for the biological and genetic independent variables used in the permutational multiple linear regression or ANOVA models. Haplotype diversity and *γ*st and values were used as the numerical dependent variable (values are reported in Table 1). Data shown are Family; Distribution range (EAM: East Africa and Madagascar; WIO: West Indian Ocean, IPO: Indo-Pacific Ocean); Mangrove habitat occupied by adult populations; Number of spawning events per year; Zone of spawning; PLD: pelagic larval duration; Av. density: average density of adult populations; Max CW: Maximum adult carapace weight; Egg female^-1^ spawning^-1^: average number of eggs produced per female per spawning event. Egg female ^-1^year^-1^: average number of eggs produced per female per year; % coverage: average percentage coverage of the vegetation belts particular to each species; Egg m^-2^ corrected: average number of eggs produced per m^2^ corrected for percentage coverage; Egg m^-2^ year^-1^ corrected: average number of eggs produced per m^2^ per year corrected for percentage coverage;; T-D: Tajima *D* test parameters. Data collected and calculated from the authors for this paper are indicated with an asterisk.
(DOCX)

S3 Table Results of the permutational multiple linear regression models performed using the biological and genetic parameters shown in S2 Table as independent variables and haplotype diversity (with and without taking into account *U*. *occidentalis*) and *γ*st as the numerical dependent variables. For each model, data shown are the values of AIC and R^2^ parameters, the number of variables involved (No. vars) and the selection of them (Selections). Variable legend: 1, Number of spawning events year ^-1^; 2, Maximum adult dimensions; 3, average population density; 5, amount of eggs m ^-2^: 6, amount of eggs female^-1^ year^-1^; 7, amount of eggs year ^-1^ m ^-2^; 8 Larval development duration (in days) and 9, Tajima D.
(DOCX)
