# Correction: Demographic History and Reproductive Output Correlates with Intraspecific Genetic Variation in Seven Species of Indo-Pacific Mangrove Crabs

**DOI:** 10.1371/journal.pone.0189550

**Published:** 2017-12-11

**Authors:** Sara Fratini, Lapo Ragionieri, Stefano Cannicci

There is an error in Table 5. The values for “Max CW (mm)” are incorrectly duplicated in all columns. Please see the corrected Table 5 here.

**Table 5:** Main life-history and ecological traits of the seven mangrove crab species.

Species	*U*. *inversa*	*U*. *occidentalis*	*U*. *hesperiae*	*P*. *guttatum*	*N*. *africanum*	*S*. *serrata*	*C*. *carnifex*	References
**Family**	Ocypodidae	Ocypodidae	Ocypodidae	Sesarmidae	Sesarmidae	Portunidae	Gecarcinidae	
**Distribution range**	EAM	WIO	WIO	EAM	EAM	IPO	IPO	[34–40]
**Mangrove habitat**	Littoral fringe	Littoral fringe	Sublittoral fringe	Eulittoral	Littoral fringe	Eulittoral/sublittoral fringe	Supralittoral	[34, 79–82]
**Spawning events year**^**-1**^	10	12	12	12	4	12	2	[23, 83–85]
**Zone of spawning**	Littoral fringe	Littoral fringe	Sublittoral fringe	Eulittoral	Littoral fringe	Oceanic platform	Sublittoral fringe	[23, 33, 83]
**PLD (days)**	26	28	26	23	29	26	25	[41, 86–91]
**Av. density (ind m**^**-2**^**)**	7.77	12.6	4.75	1.4	0.59	0.08	0.725	[82, 92, 93]
**Max CW(mm)**	20 *	17 *	25 *	30 *	42 *	150 *	100 *	/
**Egg female**^**-1**^ **spawning**^**-1**^	1600*	2400*	1750*	8200*	56700*	2000000	695000*	[85, 94, 95]

Data shown are Family; Distribution range (EAM: East Africa and Madagascar; WIO: West Indian Ocean, IPO: Indo-Pacific Ocean); Mangrove habitat occupied by adult populations; Number of spawning events per year; Zone of spawning; PLD: pelagic larval duration; Av. density: average density of adult populations; Max CW: Maximum adult carapace weight; Egg female^-1^ spawning^-1^: average number of eggs produced per female per spawning event. Data collected and calculated from the authors for this paper are indicated with an asterisk

In S2 Table, the values for “Max CW (mm)” are also incorrectly duplicated in all columns. Please view the correct S2 Table below.

## Supporting information

S2 Table Data for the biological and genetic independent variables used in the permutational multiple linear regression or ANOVA models. Haplotype diversity and *γ*st and values were used as the numerical dependent variable (values are reported in Table 1). Data shown are Family; Distribution range (EAM: East Africa and Madagascar; WIO: West Indian Ocean, IPO: Indo-Pacific Ocean); Mangrove habitat occupied by adult populations; Number of spawning events per year; Zone of spawning; PLD: pelagic larval duration; Av. density: average density of adult populations; Max CW: Maximum adult carapace weight; Egg female^-1^ spawning^-1^: average number of eggs produced per female per spawning event. Egg female ^-1^year^-1^: average number of eggs produced per female per year; % coverage: average percentage coverage of the vegetation belts particular to each species; Egg m^-2^ corrected: average number of eggs produced per m^2^ corrected for percentage coverage; Egg m^-2^ year^-1^ corrected: average number of eggs produced per m^2^ per year corrected for percentage coverage; T-D: Tajima *D* test parameters. Data collected and calculated from the authors for this paper are indicated with an asterisk.
(DOCX)
