# Supplementary material for: Demographic History and Reproductive Output Correlates with Intraspecific Genetic Variation in Seven Species of Indo-Pacific Mangrove Crabs
Source: PLoS One. 2016 Jul 5;11(7):e0158582. doi: 10.1371/journal.pone.0158582 (PMC4933389; doi:10.1371/journal.pone.0158582)
Supplement: S1 Table — For each species, the number of sequences analyzed in each locality is reported. Population groups correspond to localities as used in the statistical analyses. Sequences were obtained for this paper and from literature (see details in the text). (DOCX) [file pone.0158582.s001.docx]

S1 Table. Sampling locations

| **Group** | **Locality** | ***U. inversa*** | ***U. occidentalis*** | ***U. hesperiae*** | ***P. guttatum*** | ***N. africanum*** | ***S. serrata*** | ***C. carnifex*** |
| --- | --- | --- | --- | --- | --- | --- | --- | --- |
| **A** | Shark’s Bay, Egypt | 20 |  |  |  |  |  |  |
| **B** | Mahé Is., Seychelles |  | 8 | 19 |  |  | 26 | 15 |
| **C** | Lamu, Kenya | 20 | 4 | 20 |  | 18 | 30 | 15 |
| **D** | Mida, Kenya | 20 | 5 | 20 |  | 26 | 30 | 15 |
| **E** | Mikindani, Kenya |  | 17 |  | 16 |  |  |  |
|  | Gazi, Kenya | 20 | 12 | 21 | 10 | 27 | 30 | 15 |
|  | Shirazi, Kenya |  | 15 |  | 11 |  |  |  |
| **F** | Makoba, Tanzania |  |  |  | 13 |  |  |  |
|  | Kunduchi, Tanzania |  | 12 |  | 5 |  |  |  |
|  | Mtoni Kijichi, Tanzania |  | 9 |  | 9 |  |  |  |
|  | Ras Dege, Tanzania |  | 11 |  | 10 |  |  |  |
|  | Dar Es Salaam, Tanzania | 20 |  | 22 |  | 26 | 26 |  |
|  | Zanzibar, Tanzania |  |  |  |  |  | 20 | 15 |
| **G** | Mikindani Bay, Tanzania |  | 13 |  | 21 |  |  |  |
|  | Mnazi Bay, Tanzania |  | 10 |  | 7 |  |  |  |
|  | Ruvuma Estuary, Tanzania |  | 10 |  | 10 |  |  |  |
| **H** | Mocimboa da Praia, Mozambique |  | 19 |  | 8 |  |  |  |
|  | Ulo, Mozambique |  | 15 |  | 15 |  |  |  |
|  | Luchete, Mozambique |  | 19 |  | 10 |  |  |  |
| **I** | Ibo, Mozambique |  | 23 |  | 17 |  |  |  |
|  | Olondo, Mozambique |  | 17 |  | 22 |  |  |  |
|  | Pemba, Mozambique |  | 11 |  | 20 |  |  |  |
| **J** | Mecúfi, Mozambique |  | 24 |  | 11 |  |  |  |
|  | Nacala Velha, Mozambique |  | 16 |  | 9 |  |  |  |
|  | Cabeceira, Mozambique |  | 23 |  |  |  |  |  |
|  | Namuacha, Mozambique |  | 16 |  |  |  |  |  |
| **K** | Beira, Mozambique |  | 19 |  |  |  |  |  |
| **L** | Inkomati, Mozambique |  |  |  |  |  |  |  |
|  | Costa do Sol, Mozambique |  | 11 |  |  |  |  |  |
|  | Sangala, Mozambique |  | 20 |  |  |  |  |  |
|  | Saco, Mozambique | 20 | 11 | 23 |  | 28 | 28 |  |
|  | Ponta Rasa, Mozambique |  | 8 |  |  |  |  |  |
| **M** | Kosi Bay, South Africa |  | 18 |  |  |  |  |  |
| **N** | Richard’s Bay, South Africa |  | 8 |  |  |  |  |  |
|  | Durban, South Africa |  |  |  |  | 22 |  |  |
|  | Mlalazi Mla, South Africa |  | 17 |  |  |  |  |  |
| **TOT** |  | 120 | 421 | 125 | 224 | 147 | 190 | 75 |

For each species, the number of sequences analyzed in each locality is reported. Population groups correspond to localities as used in the statistical analyses. Sequences were obtained for this paper and from literature (see details in the text).
