# Supplementary material for: Demographic History and Reproductive Output Correlates with Intraspecific Genetic Variation in Seven Species of Indo-Pacific Mangrove Crabs
Source: PLoS One. 2016 Jul 5;11(7):e0158582. doi: 10.1371/journal.pone.0158582 (PMC4933389; doi:10.1371/journal.pone.0158582)
Supplement: S3 Table — For each model, data shown are the values of AIC and R2 parameters, the number of variables involved (No. vars) and the selection of them (Selections). Variable legend: 1, Number of spawning events year -1; 2, Maximum adult dimensions; 3, average population density; 5, amount of eggs m -2: 6, amount of eggs female-1 year-1; 7, amount of eggs year -1 m -2; 8 Larval development duration (in days) and 9, Tajima D. (DOCX) [file pone.0158582.s003.docx]

S3 Table. Results of the permutational multiple linear regression models performed using the biological and genetic parameters shown in S2 Table as independent variables and haplotype diversity (with and without taking into account *U. occidentalis*) and *γ*st as the numerical dependent variables.

| Haplotype diversity | | | | Haplotype diversity (no *U. occidentalis*) | | | | *γ*st | | | |
| --- | --- | --- | --- | --- | --- | --- | --- | --- | --- | --- | --- |
| Best result for each number of variables | | | | | | | | | | | |
| AIC | R^2^ | No. Vars | Selections | AIC | R^2^ | No. Vars | Selections | AIC | R^2^ | No. Vars | Selections |
| -27.741 | 0.476 | 1 | 3 | -45.613 | 0.908 | 1 | 5 | -67.244 | 0.392 | 1 | 9 |
| -30.236 | 0.724 | 2 | 2, 3 | -49.696 | 0.967 | 2 | 6, 7 | -76.292 | 0.875 | 2 | 7, 9 |
| -50.323 | 0.988 | 3 | 5-7 | -75.48 | 1.000 | 3 | 5-7 | -86.873 | 0.979 | 3 | 3, 7, 9 |
| -67.938 | 0.999 | 4 | 3, 5-7 | -114.25 | 1.000 | 4 | 2, 3 , 6, 9 | -93.424 | 0.994 | 4 | 2, 6, 7, 9 |
| -134.08 | 1.000 | 5 | 3, 5-8 |  |  |  |  | -130.77 | 1.000 | 5 | 5-9 |
| Overall best 5 solutions | | | | | | | | | | | |
| AIC | R^2^ | No. Vars | Selections | AIC | R^2^ | No. Vars | Selections | AIC | R^2^ | No. Vars | Selections |
| -134.08 | 1.000 | 5 | 3, 5-8 | -114.25 | 1.000 | 4 | 2, 3 , 6, 9 | -130.77 | 1.000 | 5 | 5-9 |
| -96.484 | 1.000 | 5 | 2, 3, 5-7 | -79.331 | 1.000 | 4 | 2, 3, 7, 9 | -105.52 | 0.999 | 5 | 1, 3, 6, 8, 9 |
| -83.518 | 1.000 | 5 | 2, 5-8 | -78.742 | 1.000 | 4 | 2, 5, 6, 8 | -98.569 | 0.998 | 5 | 2, 3, 6, 7, 9 |
| -79.266 | 1.000 | 5 | 1, 3, 5-7 | -78.508 | 1.000 | 4 | 2, 5, 7, 8 | -93.424 | 0.994 | 4 | 2, 6, 7, 9 |
| -71.933 | 1.000 | 5 | 3,5-7,9 | -78.407 | 1.000 | 4 | 1-3,7 | -93.387 | 0.995 | 5 | 1,5-7,9 |

For each model, data shown are the values of AIC and R^2^ parameters, the number of variables involved (No. vars) and the selection of them (Selections). Variable legend: 1, Number of spawning events year ^-1^; 2, Maximum adult dimensions; 3, average population density; 5, amount of eggs m ^-2^: 6, amount of eggs female^-1^ year^-1^; 7, amount of eggs year ^-1^ m ^-2^; 8 Larval development duration (in days) and 9, Tajima D.
