# Supplementary material for: Correction: Demographic History and Reproductive Output Correlates with Intraspecific Genetic Variation in Seven Species of Indo-Pacific Mangrove Crabs
Source: PLoS One. 2017 Dec 11;12(12):e0189550. doi: 10.1371/journal.pone.0189550 (PMC5724817; doi:10.1371/journal.pone.0189550)
Supplement: S2 Table — Haplotype diversity and γst and values were used as the numerical dependent variable (values are reported in Table 1). Data shown are Family; Distribution range (EAM: East Africa and Madagascar; WIO: West Indian Ocean, IPO: Indo-Pacific Ocean); Mangrove habitat occupied by adult populations; Number of spawning events per year; Zone of spawning; PLD: pelagic larval duration; Av. density: average density of adult populations; Max CW: Maximum adult carapace weight; Egg female-1 spawning-1: average number of eggs produced per female per spawning event. Egg female -1year-1: average number of eggs produced per female per year; % coverage: average percentage coverage of the vegetation belts particular to each species; Egg m-2 corrected: average number of eggs produced per m2 corrected for percentage coverage; Egg m-2 year-1 corrected: average number of eggs produced per m2 per year corrected for percentage coverage; T-D: Tajima D test parameters. Data collected and calculated from the authors for this paper are indicated with an asterisk. (DOCX) [file pone.0189550.s001.docx]

S2 Table. Data for the biological and genetic independent variables used in the permutational multiple linear regression or ANOVA models. Haplotype diversity and *γ*st and values were used as the numerical dependent variable (values are reported in Table 1).

| **Species** | ***U. inversa*** | ***U. occidentalis*** | ***U. hesperiae*** | ***P. guttatum*** | ***N. africanum*** | ***S. serrata*** | ***C. carnifex*** | **References** |
| --- | --- | --- | --- | --- | --- | --- | --- | --- |
| **Family** | Ocypodidae | Ocypodidae | Ocypodidae | Sesarmidae | Sesarmidae | Portunidae | Gecarcinidae |  |
| **Distribution range** | EAM | WIO | WIO | EAM | EAM | IPO | IPO | [34-40] |
| **Mangrove habitat** | Littoral fringe | Littoral fringe | Sublittoral fringe | Eulittoral | Littoral fringe | Eulittoral/sublittoral fringe | Supralittoral | [34, 79-82] |
| **Spawning events year^-1^** | 10 | 12 | 12 | 12 | 4 | 12 | 2 | [23, 83-85] |
| **Zone of spawning** | Littoral fringe | Littoral fringe | Sublittoral fringe | Eulittoral | Littoral fringe | Oceanic platform | Sublittoral fringe | [23, 33, 83] |
| **PLD (days)** | 26 | 28 | 26 | 23 | 29 | 26 | 25 | [41, 86-91] |
| **Av. density (ind m^-2^)** | 7.77 | 12.6 | 4.75 | 1.4 | 0.59 | 0.08 | 0.725 | [82, 92, 93] |
| **Max CW(mm)** | 20 * | 17 * | 25 * | 30 * | 42 * | 150 * | 100 * | / |
| **Egg female^-1^ spawning^-1^** | 1600* | 2400* | 1750* | 8200* | 56700* | 2000000 | 695000* | [85, 94, 95] |
| **Eggs female^-1^ year^-1^** | 16000* | 28800* | 21000* | 98400* | 226800* | 24000000* | 1390000* | / |
| **% coverage** | 0.45* | 0.45* | 0.45* | 0.45* | 0.45* | 0.45* | 0.1* | / |
| **Eggs m^-2^ corrected** | 2797.2* | 6804* | 1870.3* | 2583* | 7526.9* | 36000* | 25193.7* | / |
| **Eggs m^-2^ year ^-1^ corrected** | 27972* | 81648* | 22443.7* | 30996* | 30107.7* | 432000* | 50387.5* | / |
| **T-D** | -1.3* | -2.23* | -1.82* | -1.89* | -1.48* | -2.43* | -1.48* | / |

Data shown are Family; Distribution range (EAM: East Africa and Madagascar; WIO: West Indian Ocean, IPO: Indo-Pacific Ocean); Mangrove habitat occupied by adult populations; Number of spawning events per year; Zone of spawning; PLD: pelagic larval duration; Av. density: average density of adult populations; Max CW: Maximum adult carapace weight; Egg female^-1^ spawning^-1^: average number of eggs produced per female per spawning event. Egg female ^-1^year^-1^: average number of eggs produced per female per year; % coverage: average percentage coverage of the vegetation belts particular to each species; Egg m^-2^ corrected: average number of eggs produced per m^2^ corrected for percentage coverage; Egg m^-2^  year^-1^ corrected: average number of eggs produced per m^2^  per year corrected for percentage coverage; T-D: Tajima *D* test parameters. Data collected and calculated from the authors for this paper are indicated with an asterisk.
